# Willingness to vaccinate their daughters against human papillomavirus among parents of Ethiopian adolescent girls: a systematic review and meta-analysis

**DOI:** 10.1186/s40545-023-00639-9

**Published:** 2023-10-24

**Authors:** Amare Zewdie, Abebaw Wasie Kasahun, Haimanot Abebe Adane, Ayenew Mose

**Affiliations:** 1 Department of Public Health, College of Medicine and Health Science, Wolkite University, Wolkite, Ethiopia; 2 Healthy Working Lives Research Group, School of Public Health and Preventive Medicine, Monash University, Melbourne, VIC Australia; 3 Department of Midwifery, College of Medicine and Health Science, Wolkite University, Wolkite, Ethiopia

**Keywords:** Parents’ willingness, HPV vaccination, Cervical cancer, Systematic review and meta-analysis, Ethiopia

## Abstract

**Introduction:**

HPV vaccination of adolescent girls is the primary strategy for cervical cancer prevention but in Ethiopia, it lacks emphasis. Despite different studies done and found a highly variable level of parents’ willingness to vaccinate their daughter for HPV; however, there was no summarized evidence of parents' willingness as a nation. Thus this systematic review and meta-analysis aimed to assess the pooled prevalence of parents' willingness to HPV vaccination of their daughters and associated factors in Ethiopia.

**Methods:**

A systematic review and meta-analysis were conducted using PRISMA guidelines. Comprehensive literature was searched in international databases. A weighted inverse variance random effect model was used to estimate pooled prevalence. Cochrane *Q* test and *I*^2^ statistics were computed to assess heterogeneity. Funnel plot and Eggers test were done to assess publication bias. Review manager software was used to identify determinants of parents’ willingness.

**Results:**

Overall, 172 articles were retrieved and finally 7 articles were included in this review. The pooled prevalence of parents' willingness to HPV vaccination of their daughters was 71.82% (95% CI 57.73–85.91%). Knowledge about HPV vaccination (AOR = 2.80, 95% CI (2.10–3.73)), attitude (AOR = 4.93, 95% CI (3.48–6.99)), educational status (AOR = 2.19, 95% CI 1.54–3.10) and income (AOR = 3.13, 95% CI 1.96–5.02)) were significantly associated with parents' willingness.

**Conclusions:**

Parents' willingness to HPV vaccination of their daughters in Ethiopia was low. Knowledge, attitude, educational status, and income were positively associated with parents' willingness. Therefore, policymakers and program planners should target those important stakeholders (parents) in increasing their awareness and changing their attitude to enhance their vaccine acceptance specifically focusing on those who are lower in economic and educational status so as to prevent the lethal cervical cancer.

**Supplementary Information:**

The online version contains supplementary material available at 10.1186/s40545-023-00639-9.

## Introduction

Cervical cancer is a malignant tumor arising from the uterine cervix cells. It is the fourth most common cancer and cancer-related death among women globally [1]. Worldwide there were around six hundred four thousand cervical cancer cases and three hundred forty two thousand deaths in 2020 [2]. More than 85% of those affected by this cancer are young women who live in the world's poorest countries. In low- and middle-income countries its incidence is nearly twice as high and its death rate is three times as high as in high-income countries. Cervical cancer ranks second in incidence and mortality behind breast cancer in developing countries [3]. The incidence and mortality in sub-Saharan Africa are among the highest in the world and account for over 70% of the global cervical cancer burden with 70,000 new cases annually [4]. In Ethiopia, evidence shows that 35.9 new cases of cervical cancer are diagnosed, and 22.6 die from it, per 100,000 women annually [5].

Human papillomavirus (HPV) is a sexually transmitted disease, recognized as the commonest cause of cervical cancer particularly serotypes16, 18, and 31 [6]. A study done in Ethiopia also showed that HPV 16 and 31 were the most frequently detected genotypes of HPV [7]. Persistent and early undiagnosed and unmanaged HPV infection will lead to a tumorous lesion which later advances to cervical cancer which is a severe malignancy that results in death [8]. Therefore, the World Health Organization initiated multi multi-strategic approach for the prevention and control of lethal cervical cancer. One of the strategies under this approach is HPV mass vaccination of adolescent girls as the primary prevention of precancerous cervical lesions of HPV infection [9].

The first safe and efficacious HPV vaccine was developed in 2006 [10]. Evidence shows that the introduction of this HPV vaccine resulted in a reduction in persistent HPV infection and cervical lesions in several countries. HPV vaccination offers an opportunity for developing nations to decrease the burden of cervical cancer [11]. Globally, 39.7%, of women are vaccinated against HPV, while this figure is 68% in high-income countries and 2.7% in lower-middle-income countries [12]. Ethiopia introduced the HPV vaccine for girls who are 14 years of age in 2018. Then, the vaccine is delivered in a school-based approach for those target groups; however, Ethiopia as a member of low-income countries shares lower coverage of HPV vaccination [13].

Parents are the prime stakeholders in HPV vaccination for their daughters specifically mothers, since cervical cancer is a burning concern of females. Vaccine hesitancy among stakeholders at respective levels is a great challenge in the expansion of HPV as the best prevention strategy for cervical cancer. As parents are the first participants in the decision-making in the vaccination of school-age adolescent girls, they are expected to be supportive of the vaccination campaign [14, 15]. Despite no substantial adverse effects of the HPV vaccine being reported, parental misconceptions regarding the vaccine negatively affect public trust and lead to underachievement in vaccination coverage [16]. Parental willingness to vaccinate their daughters is predominantly affected by their knowledge about the disease, its consequences, level of information access, and attitude [17, 18].

Although, HPV vaccination is the primary strategy for cervical cancer prevention but in Ethiopia available research projects are focused on secondary prevention strategies, such as cervical cancer screening so, considering its importance HPV vaccination lacks emphasis. So far, in Ethiopia, different studies have been done on the willingness of parents to HPV vaccination of their daughters and found a highly variable level of willingness across the regions of the country [19–25]. Up to the level of our knowledge, there is no systematic review and meta-analysis done in Ethiopia that can provide summarized evidence on parents' willingness to HPV vaccination of their daughters; despite they are the main focus of intervention which increases the need to summarize the issue and intervene accordingly. Thus, this systematic review and meta-analysis aimed to assess the pooled prevalence of willingness to HPV vaccination and identify its associated factors among parents’ of Ethiopian adolescent girls.

### Significance of the study

The evidence from this systematic review and meta-analysis can be used for planning and implementation of an intervention to improve HPV vaccination in adolescent girls. It identifies the important factors that are associated with parental willingness; therefore, it contributes evidence input for preparing messages and materials for outreach and media campaigns to increase vaccination uptake and so as to prevent cervical cancer. This study can also serve as a baseline comparison, since there is no systematic review and meta-analysis done on this topic. In addition, it may ignite new insight for further studies that might be conducted on related topics.

## Methods

### Study design and setting

A systematic review and meta-analysis were conducted on the willingness of parents to HPV vaccination of their daughters in Ethiopia. Preferred Reporting Items for Systematic Review and Meta-Analysis guidelines were followed (Additional file 1: Table S1). PRISMA is a protocol consisting of checklists that guide the conduct and reporting of systematic reviews and meta-analyses, which increase the transparency and accuracy of reviews in medicine and other fields [26]. Ethiopia is one of the low-income countries located in the Horn of Africa with a 2022 projected population of 123.4 million [27].

### Search strategies and sources of information

We have checked the PROSPERO database (http://www.library.ucsf.edu/) whether published or ongoing projects exist related to the topic to avoid any further duplication. Thus, the findings revealed that there were no ongoing or published articles in the area of this topic. Then, this systematic review and meta-analysis was registered in the PROSPERO database with ID no of CRD42023393315. Comprehensive literature was searched using international databases PubMed, Google Scholar, and African Online Journal to retrieve-related articles from December 21 to 30, 2022. Grey literature was searched using Google. Search terms were formulated using PICO guidelines through the online databases. Medical Subject Headings (MeSH) and key terms had been developed using different Boolean operators ‘AND’ and ‘OR’. The following search term was used: “parents’ willingness” OR “parents’ acceptance” OR “parents’ readiness” OR “parents’ intention” OR “parents’ approval” AND “Human papillomavirus vaccination of their daughters” OR “Human papillomavirus vaccination of their children” AND Ethiopia.

### Eligibility criteria

To be included in this systematic review and meta-analysis, studies should be on parents' willingness to HPV vaccination of their daughters and its determinants in the English language, without restriction on race, gender, or publication date (until the last search date December 30, 2022). Parent’s willingness is defined as the proportion of participants who have positively accepted the HPV vaccination of their daughters. Therefore, included studies are expected to ask the study participants about their willingness and categorized them as willing and unwilling. Then, the response was analyzed and presented as the prevalence of parents’ willingness to HPV vaccination of their daughters. Articles without full abstracts or texts and articles reported out of the outcome interest were excluded. Citations without abstracts and/or full-text, commentaries, anonymous reports, letters, editorials, reviews, and meta-analyses were excluded at each respective stage of screening.

### Data extraction

All studies obtained from the considered databases were exported to Endnote version X8 software to remove duplicate studies. Then after, all studies were exported to a Microsoft Excel spreadsheet. All authors independently extracted the important data using a standardized data extraction form which was adapted from the Joanna Briggs Institute (JBI) data extraction format. For the first outcome (prevalence) the data extraction format included (primary author, year of publication, regions, study area, sample size, and prevalence with 95% CI). We extracted data for the second outcome (associated factors to parents’ willingness to HPV vaccination of their daughters) using a 2 by 2 table format. Finally, the log odds ratio for each factor was calculated using Review Manager (RevMan) software 5.4.

### Quality assessment

To assess the quality of each study included in this systematic review and meta-analysis, the modified Newcastle–Ottawa Quality Assessment Scale for cross-sectional studies was used [28] (Additional file 2: Table S2). Two Authors (AZ, AWK) assessed the quality of each study (i.e., methodological quality, sample selection, sample size, comparability and the outcome, and statistical analysis of the study). In the case of disagreement between two authors; another two authors (HAA, AM) were involved and discussed and resolved the disagreement.

### Data processing and analysis

The extracted Microsoft Excel spreadsheet format data were imported to STATA version 14 for analysis. Then, weighted inverse variance random effect model was used to estimate the pooled prevalence of parents’ willingness to vaccinate their daughters in Ethiopia. Cochrane *Q* test and *I*^2^ statistics were computed to assess heterogeneity among all studies. Accordingly, if the result of *I*^2^ is 0–40% it is mild heterogeneity, 40–70% would be moderate heterogeneity, and 70–100% would be considerable heterogeneity [29]. Funnel plot and Eggers test were done to assess publication bias. The *p* value > 0.05 indicated that there was no publication bias. Subgroup analysis was done based on the study region. A forest plot format was used to present the pooled prevalence of parents’ willingness to vaccinate their daughters with 95% CI. To identify determinants of parents’ willingness to HPV vaccination of their daughters, we have used review manager software.

## Results

**Fig. 1:**
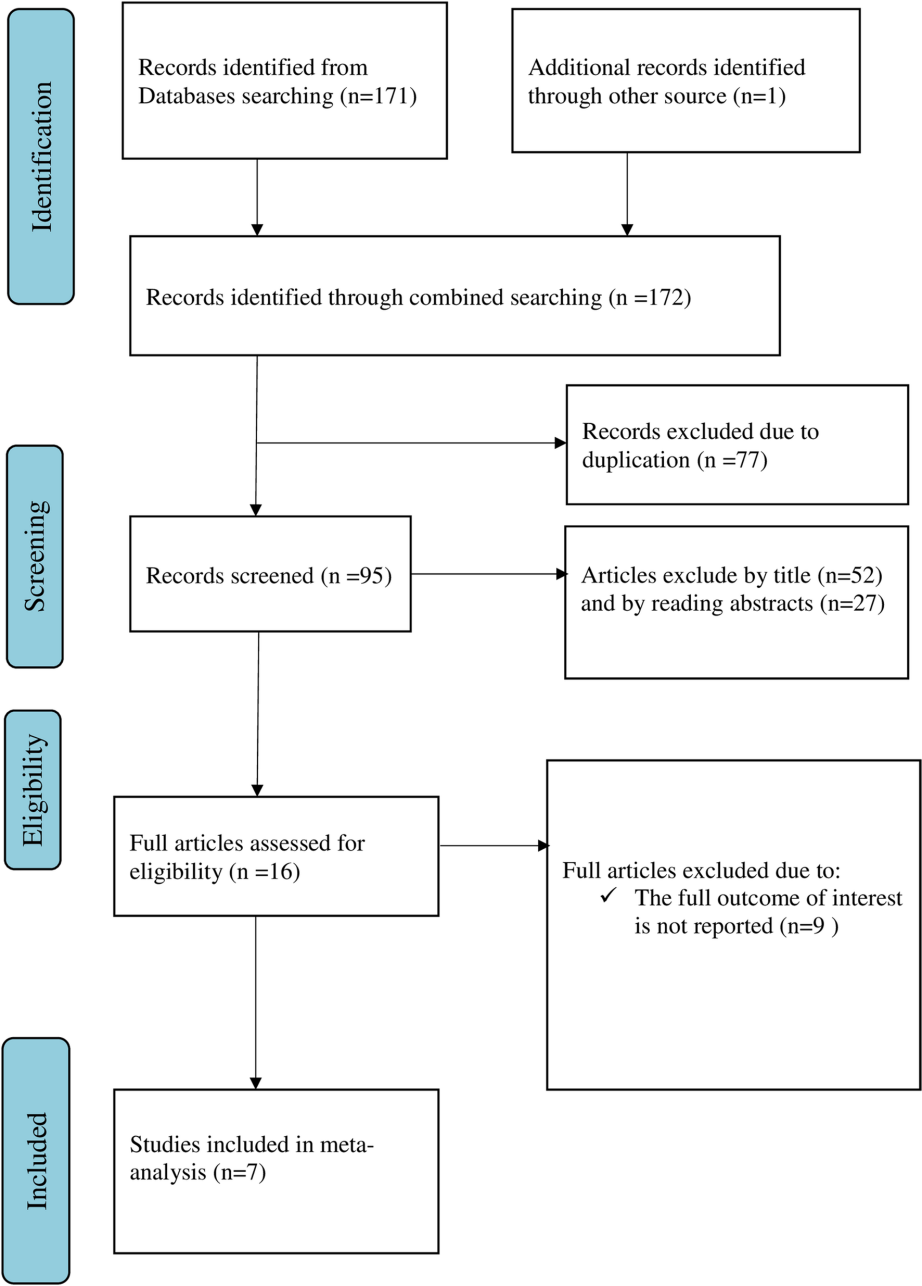
Flow chart of selection for systematic review and meta-analysis on parents’ willingness to HPV vaccination of their daughters and its determinant in Ethiopia, 2022

Overall, 172 articles were retrieved using our search strategy. International databases; PubMed, Google Scholar, and African Journals Online were searched. Duplicates (77) were removed and 95 articles remained. After reviewing, (*n* = 52) articles were excluded by title, and (*n* = 27) articles were excluded by reading abstracts. Therefore, 16 full-text articles were accessed and assessed for inclusion criteria, resulting in the further exclusion of 9 articles due to the mentioned reason. As a result, 7 studies fulfilled the inclusion criteria to undergo the final systematic review and meta-analysis (Fig. 1).

Of the included studies in this SRMA, three were done in Amhara, two in SNNPR, one in Addis Ababa, and one in the Oromia region. All included studies were cross section. They included a total of 3573 participants which ranged from 422 to 899 participants per study and found a 40.2–94.3% prevalence of willingness of parents to vaccinate their daughters. Regarding the quality of included studies, the Newcastle–Ottawa Quality Assessment scale score of all included studies lies from 8 to 9 which is good (Table 1).

**Table 1:** Characteristics of included studies in the systematic review and meta-analysis on the prevalence of willingness of parents to vaccinate their daughters and its determinants in Ethiopia

S.no	Author	Years	Region	Study design	Sample	Willingness %	Quality
1	Destaw et al. [23]	2021	SNNPR	Crossect	502	79.48	8 (good)
2	Larebo et al. [25]	2022	SNNPR	Crossect	530	84.91	8 (good)
3	Dereje et al. [19]	2021	Addis Ababa	Crossect	422	94.31	8 (good)
4	Sinshaw et al. [20]	2022	Amhara	Crossect	601	77.37	9 (good)
5	Alene et al. [24]	2020	Amhara	Crossect	899	81.31	9 (good)
6	Mihretie et al. [21]	2022	Amhara	Crossect	638	44.83	8 (good)
7	Humnesa et al. [22]	2022	Oromia	Crossect	619	40.23	8 (good)

### Magnitude of parents' willingness to HPV vaccination of their daughters in Ethiopia

**Fig. 2:**
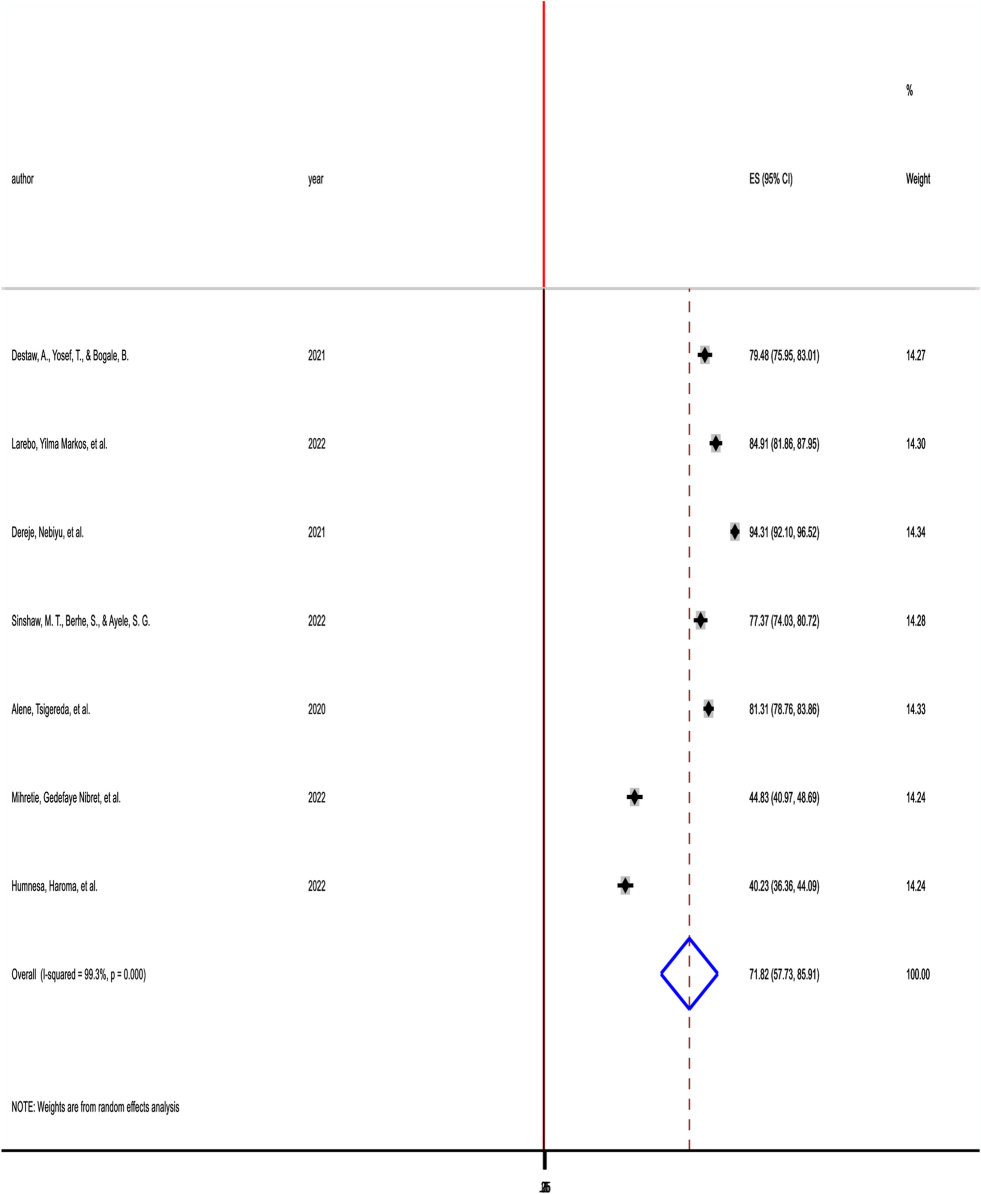
Pooled prevalence of parents’ willingness to HPV vaccination of their daughters in Ethiopia, 2022

The pooled prevalence of willingness of parents to vaccinate their daughters in Ethiopia was 71.82% (95% CI 57.73–85.91%), with the Cochrane heterogeneity index (*I*^2^ = 99.3%), *P* = 0.000, showing substantial heterogeneity of different studies (*I*^2^ > 70%). The finding was presented using a forest plot (Fig. 2).

### Publication bias 

**Fig. 3:**
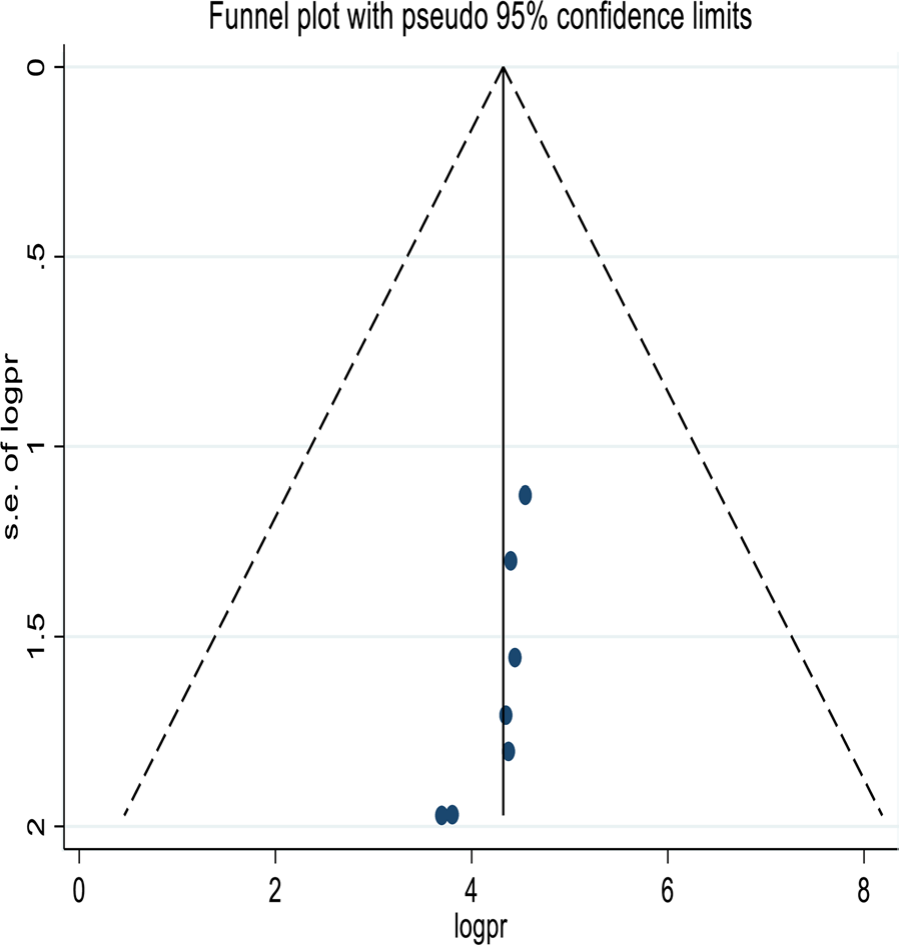
Funnel plot showing the symmetric distribution of articles on parents’ willingness to HPV vaccination of their daughters in Ethiopia, 2022

In this systematic review and meta-analysis, a funnel plot was done to check the presence of publication bias at a significance level of less than 0.05. The Egger’s regression test was not statistically significant *P* = 0.29 (*p* > 0.05) confirming no evidence of publication bias, as presented by the funnel plot (Fig. 3).

### Subgroup analysis of parents’ willingness to HPV vaccination of their daughters

**Fig. 4:**
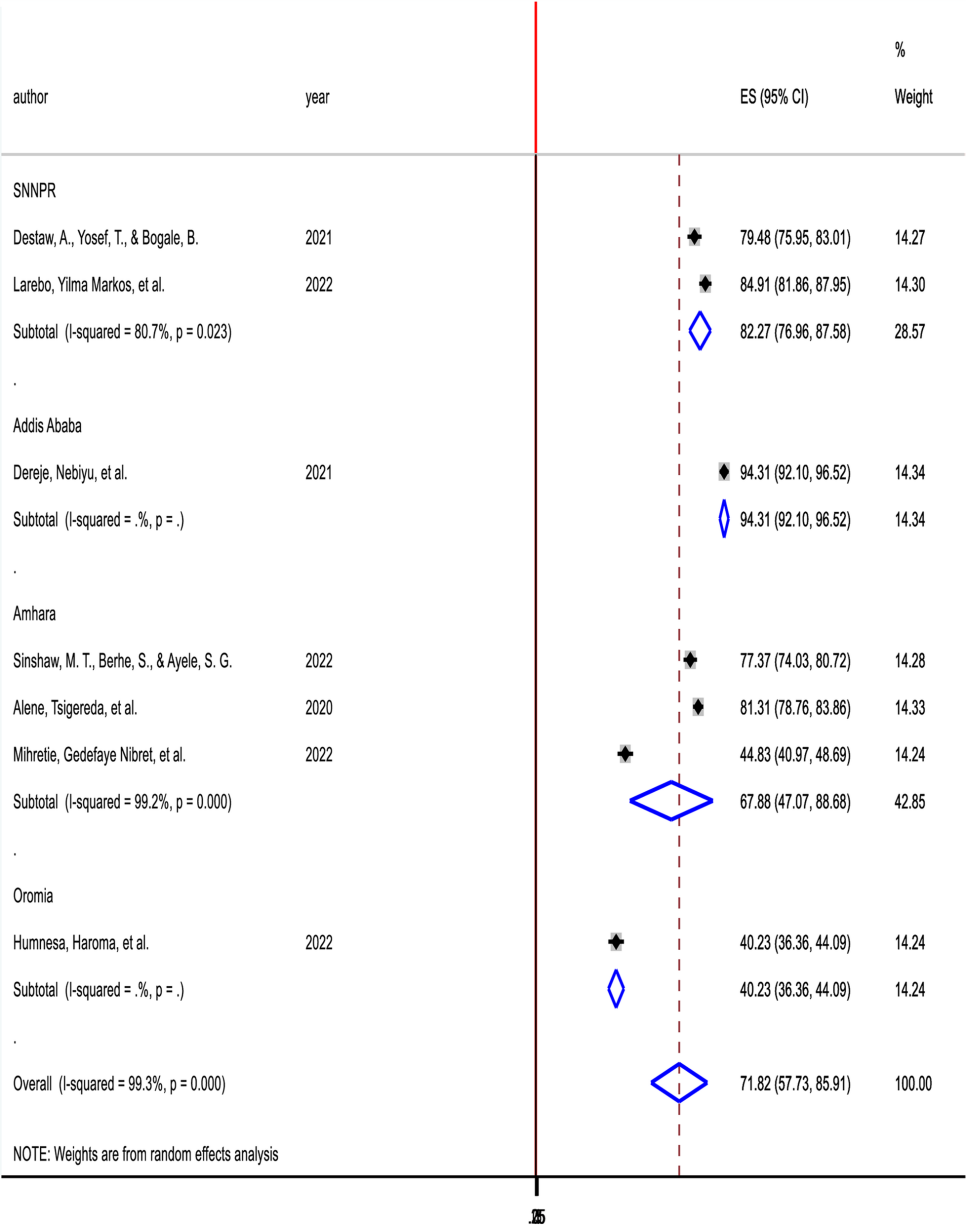
Forest plot showing Subgroup analysis of parents’ willingness to HPV vaccination of their daughters in Ethiopia, 2022

The finding of subgroup analysis by region showed that the pooled prevalence of willingness of parents to vaccinate their daughters was highest in Addis Ababa (94.31%; 95% CI (92.10–96.52), *I*^2^ = 0%, *p* = 1), followed by SNNPR (82.27%; 95% CI (76.96–87.58), *I*^2^ = 80.7%, *p* = 0.023), then Amhara (67.88%; 95% CI (47.07–88.68), *I*^2^ = 99.2%, *p* = 0.000) and least Oromia (40.23%; 95% CI (36.36–44.09), *I*^2^ = 0%, *p* = 1) (Fig. 4).

### Sensitivity analysis

**Fig. 5:**
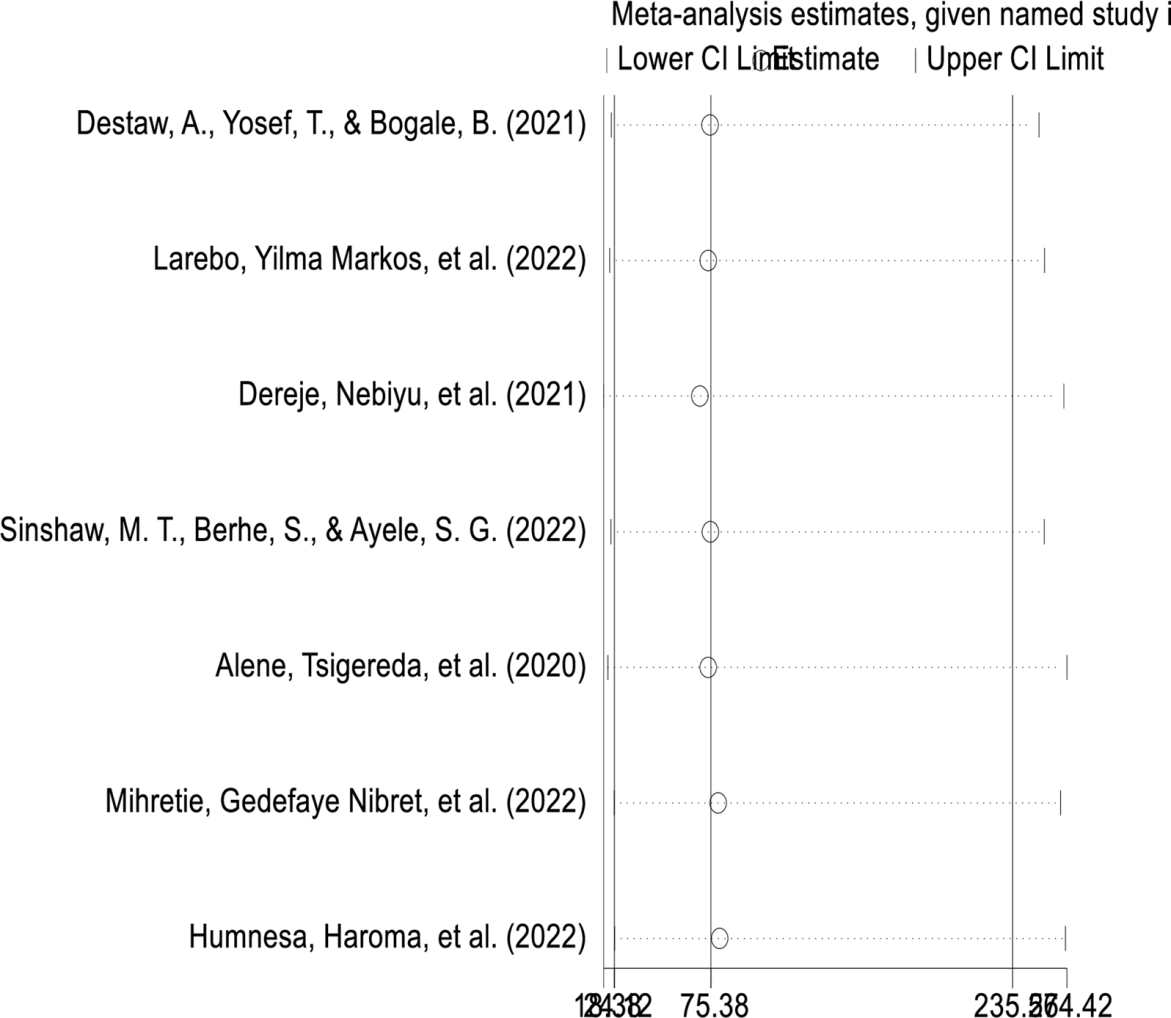
Sensitivity analysis of parents’ willingness to HPV vaccination of their daughters in Ethiopia, 2022

A random-effect model result showed that; no single study has influenced the overall pooled prevalence of willingness of parents to vaccinate their daughters in Ethiopia (Fig. 5).

### Determinants of parents’ willingness to HPV vaccination of their daughters in Ethiopia

In this systematic review and meta-analysis; four factors that are associated with parents' willingness in two or more studies are included in the identification of the determinants of willingness of parents to vaccinate their daughters. Accordingly, knowledge about HPV vaccination, attitude, educational status, and income were significantly associated with parents’ willingness to HPV vaccination of their daughters in Ethiopia. Parents who have good knowledge about the HPV vaccine were 2.80 times more likely to be willing to vaccinate their daughters as compared to their counterparts (AOR = 2.80, 95% CI (2.10–3.73)). Similarly, Parents who have a positive attitude to the vaccine were 4.93 times more likely to have a willingness to vaccinate their daughters as compared to parents who have a negative attitude (AOR = 4.93, 95% CI (3.48–6.99)). Moreover, parents having primary education and above were 2.19 times more likely to be willing to vaccinate their daughters as compared to illiterate parents (AOR = 2.19, 95% CI 1.54–3.10)). Considering income, parents from the richest household were 3.13 times more likely to be willing to vaccinate their daughters as compared to their counterparts (AOR = 3.13, 95% CI 1.96–5.02)) (Table 2).

**Table 2:** Factors associated with parents’ willingness to HPV vaccination of their daughters in Ethiopia

Variable	Authors	AOR	95% CI	Pooled AOR	95% CI of pooled AOR
Good knowledge	Mihretie et al.	3.3	2.21–4.93	2.80	2.10–3.73
	Dereje et al.	2.24	1.12–8.6		
	Destaw et al.	2.1	1.15–4.1		
	Sinshaw et al.	2.705	1.454–5.035		
Positive attitude	Dereje et al.	5.03	1.63–9.56	4.93	3.48–6.99
	Destaw et al.	2	1.3–3.41		
	Alene et al.	21.53	11.6–39.96		
Primary education and above	Mihretie et al.	1.7	1.05–2.74	2.19	1.54–3.10
	Destaw et al.	2.9	1.79–4.95		
Income	Dereje et al.	2.48	1.08–6.34	3.13	1.96–5.02
	Alene et al.	3.44	1.97–6.01		

## Discussion

In Ethiopia cervical cancer is the second most common and deadly cancer [30]. Despite WHO approved HPV mass vaccination of adolescent girls as primary prevention of precancerous cervical lesions. However, the implementation of this strategy is lower, especially in lower-income countries in which only less than 5% of adolescents are vaccinated. In Ethiopia this HPV vaccination faces several barriers. One of the barriers were lack of family support or willingness to vaccinate [31].

The current systematic review and meta-analysis aims to assess the magnitude and determinants of parents’ willingness to HPV vaccination of their daughters in Ethiopia. Accordingly, the pooled prevalence of parents’ willingness was 71.82% (95% CI 57.73–85.91%). The finding was consistent with studies done among western Nigeria (79.2%) [32], Mysore India (79.9%) [33], Bozhou China (71.5%) [34], California (75%) [35], Sweden 76% [36] and Italian parents [37]. However, the finding is lower than the study finding of Abakaliki Nigeria [38] in which 89.1% of parents of adolescent girls have willingness. The possible justification for the discrepancy might be due to the difference in the study population, since the study in Abakaliki Nigeria was conducted among the most educated individuals in which it consists 73% of the participants attended secondary education and above which increases their willingness. In addition, the Abakaliki study was conducted in a small sample size (only 290) which lowers the validity of predicted prevalence of willingness [38]. This finding implies that a strong effort at each level of the health system should be made to increase parents' willingness to enhance adolescent girls' HPV vaccination coverage to prevent cervical cancer.

The current review also identified the factor significantly associated with parents' willingness to HPV vaccination of their daughters. Thus, Parents who have good knowledge about the HPV vaccine were around three times more likely to be willing to vaccinate their daughters as compared to parents who have poor knowledge about the vaccine. The finding was similar to studies conducted in Abakaliki Nigeria [38], western Nigeria [32], and Bozhou China [34] in which parents who have better knowledge about the vaccine were more willing to vaccinate their daughters. This evidence implies respective government body’s HPV vaccination enhancement intervention should focus on improving parental awareness which can refute myths and misconceptions about the vaccine and then enhance parental acceptance and later increase vaccine uptake among adolescent girls.

In the current study favorable attitude was significantly associated with parental willingness to vaccinate their daughters for HPV. The finding was consistent with a study conducted in Poland [17], Mysore India [39], and Indonesia [40], in which favorable attitude was the statistically significant predictor of parental willingness. This may be a result of parental willingness to HPV vaccination of their daughters is mainly driven by their feelings and beliefs regarding the vaccine's effectiveness, safety, and compatibility with general societal beliefs. The finding implies the health system at each level should invest great effort to enhance parents' positive feelings and beliefs about the HPV vaccine by locally or domestically acceptable strategies regarding its safety and effectiveness.

Furthermore, in our review, parents having primary education and above were around two times more likely to have willingness to vaccinate their daughters as compared to illiterate parents. The current finding was similar to a study finding of Logos Nigeria [41] and Western Nigeria [32] in which advanced parental education was positively associated with their willingness to vaccinate their daughters. This might be due to literate parents having a chance to access better information about HPV infection and its vaccination which increases their willingness to vaccinate their daughters to prevent cervical cancer. The finding suggests that action aimed at expanding HPV vaccination should more specifically target illiterate parent to increase their awareness about the vaccine so as to enhance their acceptance.

The present review also identified that income status was a positive significant predictor of parental willingness to HPV vaccination of their daughters. This result is consistent with research findings of different studies in the world [42–44] in which parents from higher economic classes have better acceptance of the vaccine. This might be due to when the individual became economically good; he/she may have better expenditure for the promotion of their family health. Moreover, parents who had better income might have an opportunity of mass media, such as TV, radio, and other sources of information for the HPV vaccinations. This evidence calls for action that enhances HPV vaccination coverage by giving special focus to the low socio-economic population.

## Conclusion

The pooled prevalence of parents' willingness to HPV vaccination of their daughters in Ethiopia was low, which indicates that focused and carefully planned intervention should be designed and implemented to raise the level of parental willingness so as to prevent and control the lethal cervical cancer. Knowledge about HPV vaccination, attitude, educational status, and income were significantly associated with parents' willingness. Parental willingness is crucial to enhancing adolescent girls' HPV vaccination for the prevention of cervical cancer and its fatal consequences. Therefore, policymakers, program planners and the health system at each respective level should focus on improving public awareness and attitudinal change specifically targeting parents in the lower socioeconomic and education group which in turn enhances their willingness and finally increases adolescent’s vaccine uptake.

### Supplementary Information

**Additional file 1: Table S1.** PRISMA 2020 Checklist followed for this systematic review and meta-analysis.

**Additional file 2: Table S2.** Newcastle–Ottawa Quality Assessment Scale for cross-sectional studies used in the systematic review and meta-analysis 2022.

## Data Availability

The result of this SRMA was extracted from the data gathered and analyzed based on the stated methods and materials. All the relevant data are within the paper.

## Abbreviations

HPV: Human Papilloma Virus
PRISMA: Preferred reporting items for systematic reviews and meta-analyses
SNNPR: South Nation Nationality and People Region
SRMA: Systematic reviews and meta-analyses
WHO: World Health Organization
