# Supplementary material for: Willingness to vaccinate their daughters against human papillomavirus among parents of Ethiopian adolescent girls: a systematic review and meta-analysis
Source: J Pharm Policy Pract. 2023 Oct 24;16:126. doi: 10.1186/s40545-023-00639-9 (PMC10599018; doi:10.1186/s40545-023-00639-9)
Supplement: Supplementary file 2 — Additional file 2: Table S2. Newcastle–Ottawa Quality Assessment Scale for cross-sectional studies used in the systematic review and meta-analysis 2022. [file 40545_2023_639_MOESM2_ESM.docx]

| **Table S2 :** Newcastle-Ottawa Quality Assessment Scale for cross sectional studies used in the systematic review and meta-analysis 2022 | | | | | | | | |
| --- | --- | --- | --- | --- | --- | --- | --- | --- |
|  | Selection | | | | Comparability | Outcome | | Total score |
| Authors | Representativeness s (1) | Sample size (1) | Non respondents (1) | Ascertainment of the exposure (risk factor) (2) | The subjects in different outcome groups are comparable, based on the study design or analysis. confounding factors are controlled (2) | Assessment of the outcome (2) | Statistical test (1) |  |
| Destaw, A., Yosef, T., & Bogale, B. | 1 | 1 | 1 | 1 | 1 | 2 | 1 | 8 |
| Larebo, Yilma Markos, et al. | 1 | 1 | 1 | 2 | 1 | 1 | 1 | 8 |
| Dereje, Nebiyu, et al. | 1 | 1 | 1 | 2 | 1 | 1 | 1 | 8 |
| Sinshaw, M. T., Berhe, S., & Ayele, S. G. | 1 | 1 | 1 | 2 | 1 | 2 | 1 | 9 |
| Alene, Tsigereda, et al. | 1 | 1 | 1 | 2 | 1 | 2 | 1 | 9 |
| Mihretie, Gedefaye Nibret, et al. | 1 | 1 | 1 | 1 | 1 | 2 | 1 | 8 |
| Humnesa, Haroma, et al. | 1 | 1 | 1 | 2 | 1 | 1 | 1 | 8 |
